# Genetic prediction of male pattern baldness

**DOI:** 10.1371/journal.pgen.1006594

**Published:** 2017-02-14

**Authors:** Saskia P. Hagenaars, W. David Hill, Sarah E. Harris, Stuart J. Ritchie, Gail Davies, David C. Liewald, Catharine R. Gale, David J. Porteous, Ian J. Deary, Riccardo E. Marioni

**Affiliations:** 1 Centre for Cognitive Ageing and Cognitive Epidemiology, University of Edinburgh, Edinburgh, United Kingdom; 2 Department of Psychology, University of Edinburgh, Edinburgh, United Kingdom; 3 Division of Psychiatry, University of Edinburgh, Edinburgh, United Kingdom; 4 Centre for Genomic and Experimental Medicine, Institute of Genetics and Molecular Medicine, University of Edinburgh, Edinburgh, United Kingdom; 5 Medical Research Council Lifecourse Epidemiology Unit, University of Southampton, Southampton, United Kingdom; University of Bonn, GERMANY

## Abstract

Male pattern baldness can have substantial psychosocial effects, and it has been phenotypically linked to adverse health outcomes such as prostate cancer and cardiovascular disease. We explored the genetic architecture of the trait using data from over 52,000 male participants of UK Biobank, aged 40–69 years. We identified over 250 independent genetic loci associated with severe hair loss (P<5x10^-8^). By splitting the cohort into a discovery sample of 40,000 and target sample of 12,000, we developed a prediction algorithm based entirely on common genetic variants that discriminated (AUC = 0.78, sensitivity = 0.74, specificity = 0.69, PPV = 59%, NPV = 82%) those with no hair loss from those with severe hair loss. The results of this study might help identify those at greatest risk of hair loss, and also potential genetic targets for intervention.

## Author summary

Living with male pattern baldness can be stressful and embarrassing. Previous studies have shown baldness to have a complex genetic architecture, with particularly strong signals on the X chromosome. However, these studies have been limited by small sample sizes. Here, we present the largest genome-wide study of baldness to date, using data from over 52,000 male participants in the UK Biobank study. We identify over 200 novel findings. We also split our dataset in two to build and apply a genetic predictor of baldness. Of those with a polygenic score below the median, 14% had severe hair loss and 39% no hair loss. By contrast, of those with a polygenic score in the top 10%, 58% reported moderate-to-severe hair loss.

## Introduction

Male pattern baldness affects around 80% of men by the age of 80 years [1], and it can have substantial psychosocial impacts via changes in self-consciousness and social perceptions [2, 3]. In addition to alterations in physical appearance, some, but not all, studies have identified negative health outcomes associated with baldness including increased risk of prostate cancer [4–6] and cardiovascular disease [7–9]. Baldness is known to be substantially heritable [10]. Here, we use a large, population-based dataset to identify many of the genes linked to variation in baldness, and build a genetic score to improve the prediction of severe hair loss.

The total proportion of variance in male pattern baldness that can be attributed to genetic factors has been estimated in twin studies to be approximately 80% for both early- and late-onset hair loss [11, 12]. Newer molecular-genetic methods have estimated the single-nucleotide polymorphism (SNP)-based, common-variant heritability of baldness at around 50% [13]. Molecular methods also indicate a degree of overlap between genetic variants linked to baldness and those linked to phenotypes such as height, waist-hip ratio, age at voice drop in males, age at menarche in females, and presence of a unibrow [14].

A number of studies have identified specific genetic variants linked to variations in baldness, usually with the *AR* gene showing the strongest association. The largest published genome-wide association study (GWAS) to date highlighted eight independent genetic loci that were linked to baldness; the top *AR* SNP yielded an odds ratio of 2.2 in a case-control meta-analysis of 12,806 individuals of European ancestry [15]. One of the autosomal hits identified in that study was found to be in a gene linked to Parkinson’s disease. More recently, a review paper highlighted fifteen loci from six studies that have been associated at genome-wide significance (P<5x10^-8^) with baldness; two of these were located on the X chromosome [16].

Several attempts have been made to build predictors of male pattern baldness using polygenic risk scores. Heilmann et al. found, using a case-control design with ~600 per arm, that a predictor based on 34,186 SNPs explained 4.5% of the variance on the liability scale [17]. Marcińska et al. used candidate genes to build 5-SNP and 20-SNP polygenic predictors, which performed well when considering prediction of early-onset male pattern baldness, but poorly when considering those with no baldness versus those with severe baldness across all ages [18]. Most recently, a 20-SNP predictor was assessed in three European studies [13]. It achieved a maximum Area Under the Curve (AUC) prediction of 0.74 in an early-onset cohort, but weaker estimates in the other two, late-onset cohorts (AUC = 0.69 and 0.71). These values correspond to poor-to-fair predictions of baldness. In addition, in that study, age was included in the predictor, explaining the bulk of the differences. A meta-analysis of the three cohorts’ GWAS studies identified a novel locus on chromosome 6. The study also estimated the SNP-based heritability of early-onset (56% (SE 22%) from the autosomes, 23% (SE 1.1%) from the X chromosome) and late-onset baldness (42% (SE 23%) from the autosomes, 10% (SE 5%) from the X chromosome).

### The present study

The UK Biobank study [19] (http://www.ukbiobank.ac.uk) is a large, population-based genetic epidemiology cohort. At its baseline assessment (2006–2010), around 500,000 individuals aged between 40 and 70 years and living in the UK completed health and lifestyle questionnaires and provided biological samples for research.

The present study reports a GWAS of male pattern baldness in the UK Biobank cohort, which is over four times the size of the previously-largest meta-analytic study [15]. After completing the GWAS, we split the cohort into a large ‘discovery’ sample of 40,000 participants in which the GWAS was re-run. The regression weights from this GWAS were used to perform a prediction analysis in the sub-sample of 12,000 participants who did not contribute to the GWAS. We determined the accuracy of the polygenic profile score by discriminating between those with severe hair loss and those with no hair loss.

## Results

The mean age of the 52,874 men was 57.2 years (SD 8.0). 16,724 (31.6%) reported no hair loss, 12,135 (23.0%) had slight hair loss, 14,234 (26.9%) had moderate hair loss, and 9,781 (18.5%) had severe hair loss.

**Fig 1:**
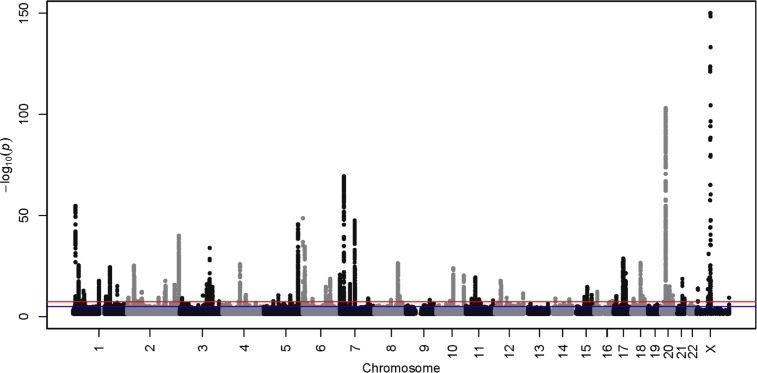
Manhattan Plot of imputed autosomal GWAS and genotyped X chromosome of male pattern baldness (p-values truncated at 1x10^-150^).

The genome-wide association study of the four-category self-reported baldness measure in 52,874 White British men from UK Biobank yielded 13,029 autosomal hits from the imputed data (P<5x10^-8^), in addition to 117 hits (out of 14,350 genotyped SNPs) on the X chromosome (**Fig 1**). The QQ plot for the autosomal GWAS is shown in **S1 Fig**. An LD clumping analysis indicated that these hits can be attributed to 247 independent autosomal regions. All previously reported autosomal hits [10, 13–16] that mapped to SNPs in our study (62 out of 68 SNPs) replicated with a maximum P-value of 0.006 (54 out of 62 lookups had P<5x10^-8^, **S1 Table**). The previously reported X chromosome variant from Li et al. [15] and the variant from Richards et al. [10] also replicated with P-values that were effectively zero (**S1 Table**). The chromosome 6 hit (rs4959410) from Liu et al. [13], which was not supported by additional SNPs in the region, failed to replicate (P = 0.37). All other hits from Liu et al. [13] had been previously reported in the literature. A list of the top 20 independent autosomal hits are presented in **Table 1**. The top 10 independent X chromosome hits are presented in **Table 2**; rs140488081 and rs7061504 are intronic SNPs in the *OPHN1* gene. After conditioning on the top SNP (rs73221556), 47 SNPs (including the two lead X chromosome SNPs from the literature: rs2497938 and rs6625163) remained significant at P<5x10^-8^. In the UK Biobank data, the two lead SNPs from the literature were in very high LD (R^2^ = 0.98). Summary output for all of the SNPs is available at the following URL: http://www.ccace.ed.ac.uk/node/335. A list of the 287 independent loci are reported in **S2 Table**.

**Table 1:** Top 20 independent autosomal GWAS hits.

Chr	Position	SNP ID	Effect allele	MAF	Beta	SE	P	SNPs in Clump	SNPs with P<0.0001	Gene	Function
1	11040385	rs7542354	A	0.22	-0.12	0.007	5.74 x10^-55^	86	80	*C1orf127*	intronic
1	25467880	rs12745121	A	0.31	-0.07	0.007	5.51 x10^-26^	134	56	NA	NA
1	170361164	rs10919382	G	0.38	0.07	0.006	4.54 x10^-25^	514	463	NA	NA
2	32181424	rs13021718	A	0.14	-0.09	0.009	7.26 x10^-26^	541	396	*MEMO1/DPY30*	intronic
2	239695893	rs11684254	G	0.35	0.09	0.006	1.10 x10^-40^	140	134	AC144525.1	3downstream
3	139032333	rs7642536	C	0.14	0.11	0.009	1.28 x10^-34^	49	40	*MRPS22*	intronic
4	81197949	rs7680591	A	0.42	0.07	0.006	1.44 x10^-26^	107	103	*FGF5*	intronic
5	158320877	rs1422798	G	0.38	-0.09	0.006	2.84 x10^-46^	261	176	*EBF1*	intronic
6	396321	rs12203592	T	0.22	0.11	0.007	2.64 x10^-49^	8	8	*IRF4*	intronic/5upsteam
6	9327556	rs9357047	C	0.44	0.08	0.006	3.07 x10^-35^	229	194	NA	NA
7	18896988	rs71530654	G	0.40	0.11	0.006	4.68 x10^-70^	98	98	*HDAC9*	intronic
7	68587797	rs939963	C	0.45	-0.09	0.006	3.58 x10^-48^	104	94	NA	NA
8	109145555	rs79206101	T	0.01	0.31	0.028	4.91 x10^-27^	25	24	AP001331.1	5upstream
17	44066172	rs112385572	G	0.24	-0.08	0.007	8.45 x10^-29^	376	366	*MAPT*	intronic
17	44787313	rs538628	C	0.22	-0.08	0.007	3.60 x10^-27^	81	77	*NSF*	intronic
18	42814156	rs8085664	A	0.28	-0.07	0.007	3.47 x10^-27^	338	306	*SLC14A2*	intronic
20	21894764	rs6035986	T	0.43	0.12	0.006	1.20 x10^-87^	0	0	NA	NA
20	22033819	rs201593	A	0.43	0.13	0.006	1.06 x10^-103^	662	662	*LOC100270679/ RP11-125P18*.*1*	5upstream
20	22100070	rs7362397	T	0.30	-0.11	0.007	3.02 x10^-65^	0	0	NA	NA
20	22100072	rs7362398	T	0.30	-0.11	0.007	3.02 x10^-65^	0	0	NA	NA

**Table 2:** List of top 10 genotyped male pattern baldness GWAS hits for the X chromosome.

Chr	BP	SNP ID	Effect Allele	Beta	SE	P	SNPs in clump	SNPs with P<0.0001
X	65933285	rs73221556	A	-0.53	0.02	<5.1 x10^-178^	19	19
X	66481800	rs12558842	C	-0.54	0.03	<5.1 x10^-178^	8	8
X	67003584	rs5919427	C	-0.35	0.02	5.1 x10^-178^	11	11
X	67496002	rs140488081	T	-0.40	0.01	4.6 x10^-61^	9	4
X	65083247	rs147154263	T	-0.43	0.02	3.6 x10^-58^	2	2
X	67139063	rs148652266	A	-0.51	0.01	7.7 x10^-45^	1	1
X	65541956	rs145867342	T	-0.32	0.01	1.1 x10^-44^	2	2
X	67363801	rs7061504	G	0.19	0.04	1.7 x10^-38^	5	4
X	58005480	rs147829649	G	-0.28	0.01	1.3 x10^-31^	1	0
X	66337545	rs17216820	T	0.19	0.02	5.8 x10^-26^	2	2

The gene-based analysis identified 112 autosomal genes and 13 X chromosome genes that were associated with baldness after a Bonferroni correction (P<0.05/18,061 and P<0.05/567, respectively). The top gene-based hit was, as expected, the androgen receptor on the X chromosome (P = 2.0x10^-269^). A full list of the autosomal significant gene-based hits is provided in **S3 Table** and significant genes on the X chromosome are shown in **S4 Table**. A significant enrichment (FDR <0.05) was found for 143 gene sets; the full results are presented in **S5 Table**.

Using common genetic variants with a minor allele frequency of at least 1%, GCTA-GREML analysis found that 47.3% (SE 1.3%) of the variance in baldness can be explained by common autosomal genetic variants, while 4.6% (SE 0.3%) can be explained by common X chromosome variants.

Genetic correlations were examined between male pattern baldness and 24 cognitive, health, and anthropometric traits using LD Score regression. No significant associations were found; all estimates were close to zero (**S6 Table**).

**Fig 2:**
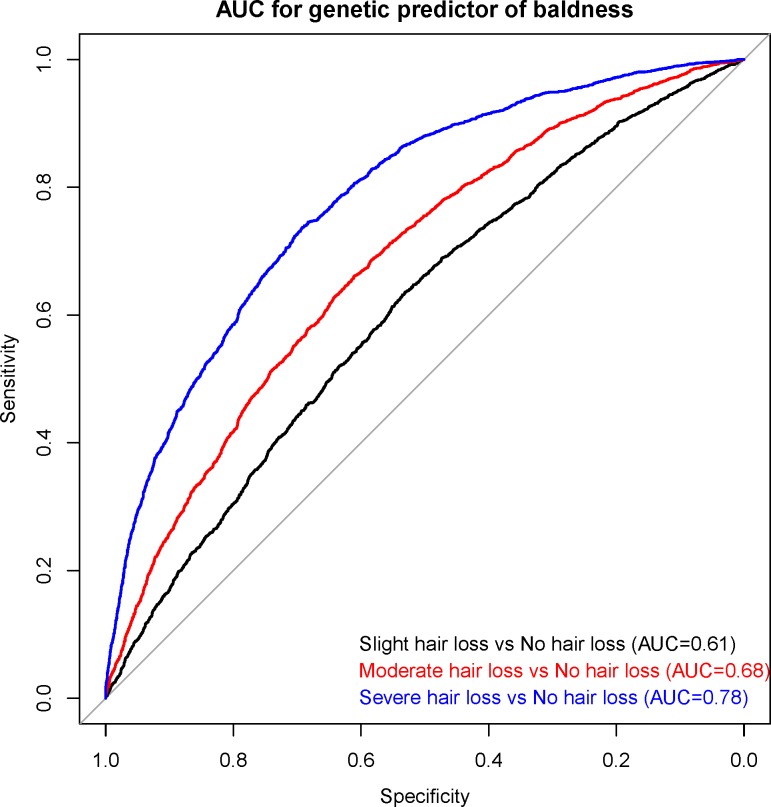
Area under the curve plot for discriminating those with hair loss from those with no loss.

**Fig 3:**
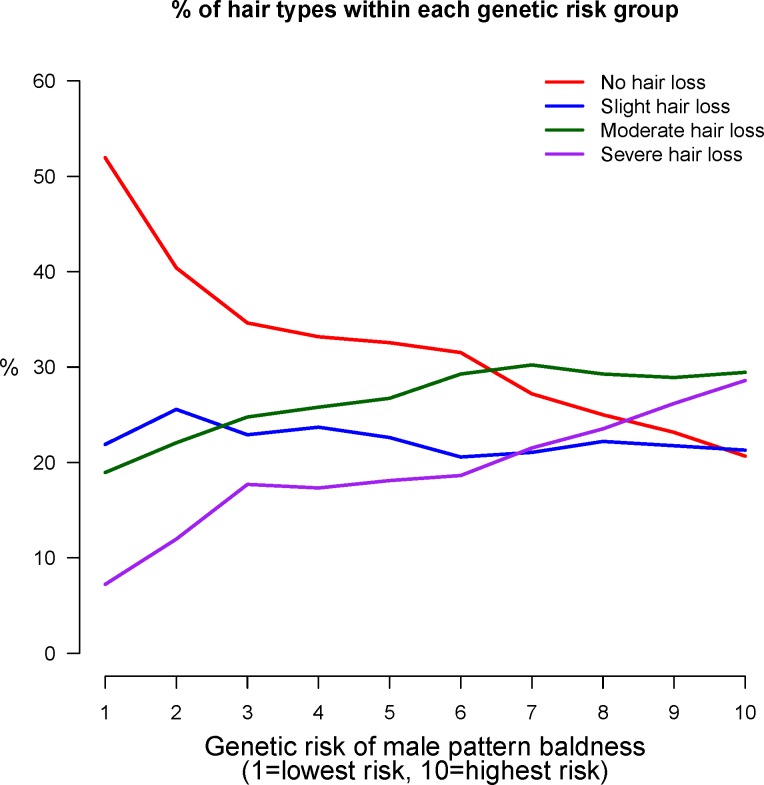
Distribution of hair loss by male pattern baldness polygenic score decile in the independent sample.

The GWAS for self-reported baldness was re-run on a sub-sample of 40,000 individuals—retaining an equal proportion of each of the four baldness patterns as observed in the full cohort—to allow a polygenic prediction score to be built and applied to the remaining, independent sample of 12,874 individuals. The most powerful predictions from comparing the extreme phenotype groups were observed at the P<1x10^-5^ threshold for both the autosomal and X chromosome polygenic scores (**Table 3**). The optimal autosomal polygenic score yielded an AUC of 0.75 for discriminating between those with no hair loss (n = 4,123) and those with severe hair loss (n = 2,456). The corresponding AUC for the optimal X chromosome polygenic score was 0.62. An additive combination of the autosomal and X chromosome polygenic scores gave an AUC of 0.78 (sensitivity = 0.74, specificity = 0.69, PPV = 0.59, NPV = 0.82) for severe hair loss, 0.68 (sensitivity = 0.66, specificity = 0.61, PPV = 0.58, NPV = 0.68) for moderate hair loss, and 0.61 (sensitivity = 0.64, specificity = 0.53, PPV = 0.49, NPV = 0.68) for slight hair loss (**Fig 2**). Adding age as a covariate boosted the AUC to 0.79 for severe hair loss (P<2x10^-16^), 0.70 for moderate hair loss (P<2x10^-16^), and 0.61 for slight hair loss (P = 0.019). **Fig 3** shows the proportion of participants in the four baldness groups for each polygenic risk decile of male pattern baldness. Of those with a baldness polygenic score below the median, 14% reported severe hair loss and 39% no hair loss. By contrast, of those with a polygenic score in the top 10%, 58% reported moderate-to-severe hair loss.

**Table 3:** AUC results for severe hair loss versus no hair loss for all autosomal and X chromosome polygenic thresholds.

Genomic region	P value threshold	n SNPs	AUC
Autosomal	<1x10^-20^	11	0.648
	<5x10^-8^	107	0.725
	<1x10^-5^	261	0.748
	<0.01	7365	0.725
	<0.05	28097	0.701
	<0.10	51178	0.687
	<0.50	205679	0.663
	<1	346958	0.662
X chromosome	<1x10^-20^	16	0.606
	<5x10^-8^	44	0.612
	<1x10^-5^	70	0.621
	<0.01	284	0.619
	<0.05	785	0.618
	<0.10	1329	0.615
	<0.50	4746	0.611
	<1	7989	0.611

The results of the partitioned heritability analysis indicated that 27 of the functional annotations from the baseline model were statistically significant (**S2 Fig** and **S7 Table**). These significant annotations included a broad array of functional elements including histone marks, enhancer regions, conserved regions, and DNaseI hypersensitivity sites (DHS). The ten tissue types were then tested for significance after controlling for the baseline model. Following correction for multiple testing, all ten of the tissue groups showed significant enrichment (**S3 Fig** and **S7 Table**).

## Discussion

In this large GWAS study of male pattern baldness, we identified 287 independent genetic signals that were linked to differences in the trait, a substantial advance over the previous largest GWAS meta-analysis, which identified eight independent signals [15]. We showed—in line with a previous study [13], but with much greater precision—that a substantial proportion of individual differences in hair loss patterns can be explained by common genetic variants on the autosomes as well as on the X chromosome. However, the variance explained by X chromosome variants is much lower for late-onset compared to early-onset male pattern baldness [13]. Finally, by splitting our cohort into a discovery and a prediction sample, we showed a predictive discrimination (AUC = 0.78) between those with no hair loss and those with severe hair loss.

Despite there being genetic overlap for SNP hits associated with baldness and Parkinson’s Disease—first noted in Li et al. [15] and replicated here—we observed no statistically significant genetic correlations after correcting for multiple testing between baldness and any of the health, cognitive, or anthropometric outcomes we studied. There were very small (maximum absolute genetic correlation of 0.13) but nominally-significant associations with height, bipolar disorder, number of children born, and age at menarche, such that the genes associated with more hair loss were linked to shorter stature, younger age at menarche, fewer offspring, and a lower risk of bipolar disorder (all P<0.05). The local but not global overlap of SNPs associated with baldness and other traits, such as Parkinson's Disease, might be explained by chance due to the large number of hits for baldness, or pleiotropy at single sites with no systemic overlap. The point estimates for the genetic correlations were all near zero, suggesting true null associations as opposed to a lack of statistical power to detect modest-sized correlations.

As mentioned above, the GWAS identified 247 independent autosomal loci and 40 independent X chromosome loci. The top 20 hits from the autosomes were located in or near to genes that have been associated with, for example, hair growth/length in mice (*FGF5*) [20], grey hair (*IRF4*) [21], cancer (breast: *MEMO1* [22], bladder: *SLC14A2* [23]), histone acetylation (*HDAC9*), and frontotemporal dementia (*MAPT*) [24]. A previous GWAS showed an association of *IRF4* with both hair colour and hair greying, but not with male pattern baldness [21]. *HDAC9* has been identified as a baldness susceptibility gene in a previous study [25]. Two of the top 10 X chromosome SNPs were located in *OPHN1*, a gene previously associated with X-linked mental retardation [26].

Of the top autosomal gene-based findings (maximum P = 3.1x10^-15^), *RSPO2* has been linked to hair growth in dogs. *PGDFA* has been linked to hair follicle development [27]; *EBF1* is expressed in dermal papillae in mature hair follicles [28]; *PRR23B* is proximal to a GWAS hit for eyebrow thickness [21]; and *WNT10A* has been linked to both straight hair [29] and dry hair [30]. The WNT signaling pathway is involved in the activation of β-catenin, which regulates the differentiation of follicular keratinocytes, which form the hair follicle [31].

The top X chromosome gene-based findings included the androgen receptor (*AR*), which has been well established as a baldness associated gene [32], along with its upstream (*EDA2R*) and downstream (*OPHN1*) genes. *EDA2R* plays a role in the maintenance of hair and teeth as part of the tumor necrosis factor receptor. Onset of male pattern baldness could be influenced by *EDA2R* via activation of nuclear proto-oncoprotein *c-Jun*, which is linked to transcription activation of *AR* [33]. Two other genes included in the gene-based findings, *OPHN1* and *ZC4H2*, have previously been associated with X-linked mental retardation [26, 34]. One limitation of our X chromosome analysis was that it contained genotyped SNPs only. The imputed X chromosome SNP data for UK Biobank have not yet been released but will likely provide further clues about the genetic architecture of male pattern baldness.

Many of the genes identified are associated with hair structure and development, which may be critical for the process of hair loss. For example, animal models indicate that FGF5 is critical for the inhibition of hair growth and mutations in *FGF5* are associated with excessively long eyelashes in humans [35]. It is possible that genetic variants leading to higher levels of expression of this gene result in greater inhibition of hair growth, leading to male pattern baldness. As a second example, the *RSPO2* gene is associated with hair growth in dogs [36]. It is part of the *Wnt* signalling pathway needed for the establishment of hair follicles [37]. Variation in the activity of this pathway caused by genetic variants within *PSPO2* may lead to differences in levels of hair growth in men and may contribute to male pattern baldness. The inclusion of hits on the X chromosome, specifically the Androgen Receptor, suggests that hormonal mechanisms are also involved in hair loss. It is possible that the hair structure proteins interact biologically with sex hormones, leading to a higher prevalence of baldness.

The results of the gene set analysis indicated that the genomic regions with the greatest evidence for association with male pattern baldness are united by a shared biological theme. In particular, these associated regions appear to converge on the transcription factor complex, and transcription factor binding gene sets.

The most significant gene set, GO:0005667, corresponded to the transcription factor complex gene set, which includes the gene *ALX4*. *ALX4* was found to be mutated in a patient with frontonasal dysplasia, presenting with alopecia [38]. Of the other genome-wide significant gene sets, ENSG00000141027 (NCOR1 subnetwork), includes members of the histone deacetylase (HDAC) family [39]. *HDAC9* is associated with male pattern baldness (the present paper and Li et al. [15]). GO:0003682 (transcription factor binding), includes the murine gene *Cux1* that is important for, amongst other things, hair growth [40]. GO:0003712 (transcription cofactor activity), includes the gene *AIRE*, which is associated with alopecia [41]. MP:0000097, (short maxilla), and GO:0044212 (transcription regulatory region DNA binding), both include the murine gene *Grhl1*. Grhl1-null mice suffer from a delay in coat growth and later hair loss [42]. It is important to note that, as with all pathway analyses, the results are dependent on the gene sets defined in the databases used. These rely on accurate functional annotations, which are continually updated.

The main strength of this study is its large sample size and phenotypic homogeneity. Many meta-analytic studies of complex traits are weakened by different cohorts collecting data at different time-points, under different protocols, in different populations. The present study replicated all of the previously identified autosomal hits for baldness from Li et al. [15] and Heilmann-Heimbach et al.,[16] suggesting a degree of robustness in phenotypic measurement, which was briefer here than in previous studies of male pattern baldness. Whereas the genomic inflation factor from the GWAS was large (1.09, Q-Q plot in **S1 Fig**), this is likely to be a result of genuine polygenic effects. We have used identical analysis protocols for other traits with far lower SNP-based heritabilities in the same UK Biobank cohort and observed no evidence of inflation [43].

### Conclusion

We identified over two hundred independent, novel genetic correlates of male pattern baldness—an order of magnitude greater than the list of previous genome-wide hits. Our top SNP and gene-based hits were in genes that have previously been associated with hair growth and development. We also generated a polygenic predictor that discriminated between those with no hair loss and those with severe hair loss. Whereas accurate predictions for an individual are still relatively crude, of those with a genetic score in the top 10% of the distribution, 58% reported moderate-to-severe hair loss. The release of genetic data on the full UK Biobank cohort will further refine these predictions and increase our understanding of the genetic architecture of male pattern baldness.

## Methods

### Data

Data came from the first release of genetic data of the UK Biobank study and analyses were performed under the data application 10279. Ethical approval for UK Biobank was granted by the Research Ethics Committee (11/NW/0382).

### Genotyping information

Genotyping details including quality control steps have been reported previously [43]. Briefly, from the sample with genetic data available as of June 2015, 112,151 participants remained after the following exclusion criteria were applied: SNP missingness, relatedness, gender mismatch, non-British ancestry, and failed quality control for the UK BiLEVE study [43]. For the current analysis, an imputed dataset was used for the autosomes (reference set panel combination of the UK10K haplotype and 1000 Genomes Phase 3 panels: http://biobank.ctsu.ox.ac.uk/crystal/refer.cgi?id=157020). Imputed data were not available for the X chromosome, hence only genotyped variants were considered. X chromosome quality control steps included a minor allele frequency cut-off of 1% and a genotyping call rate cut-off of 98% [44]. For the imputed autosomal data, we restricted the analyses to variants with a minor allele frequency >0.1% and an imputation quality score >0.1.

### Male pattern baldness phenotype

**Fig 4:**
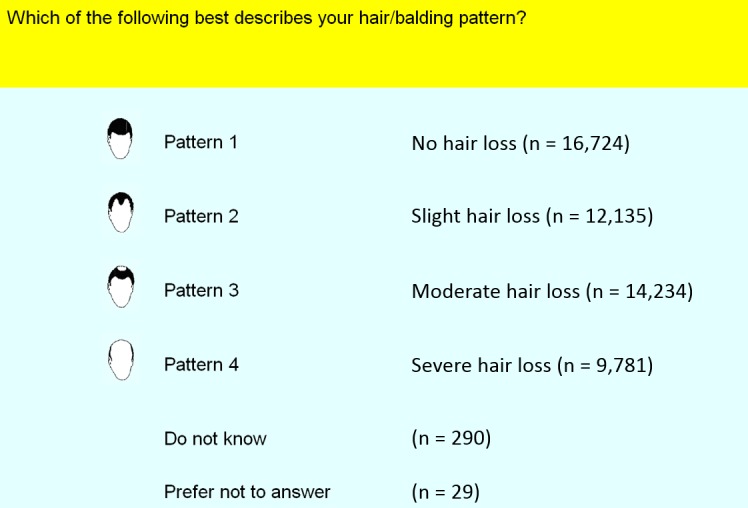
Screenshot of UK Biobank question 2395 on male pattern baldness, adapted with permission.

From the sample of 112,151 unrelated White British participants with genetic data, we identified 52,874 men with a self-reported response to UK Biobank question 2395, which was adapted from the Hamilton-Norwood scale [45, 46]. These men were asked to choose, from four patterns (no loss; slight loss; moderate loss; severe loss), the one that matched their hair coverage most closely. **Fig 4** shows a screenshot of the four options.

### GWAS of male pattern baldness on the whole sample

A genome-wide association study was conducted using baldness pattern residuals as the dependent variable. The residuals were obtained from a linear regression model of baldness pattern on age, assessment centre, genotyping batch and array, and 10 principal components to correct for population stratification.

The GWAS for the imputed autosomal dataset was performed in SNPTest v2.5.1 [47] via an additive model, using genotype probability scores. The GWAS for the X chromosome was performed in Plink [48, 49].

### Identification of independent GWAS signals

The number of independent signals from the GWAS was determined using LD-clumping [48, 49] based on the LD structure annotated in the 1000 genomes project [50]. Index SNPs were identified (P<5x10^-8^) and clumps were formed for SNPs with P<1x10^-5^ that were in LD (R^2^>0.1) and within 500kb of the index SNP. SNPs were assigned to no more than one clump.

### Lookup of published male pattern baldness hits

GWAS lookups were performed for the top hits reported in Richards et al. [10], Li et al. [15], Heilmann-Heimbach et al. [16], Liu et al. [13] and Pickrell et al. [14].

### Gene-based correlates of male pattern baldness

Gene-based analyses were performed using MAGMA [51]. SNPs were mapped to genes according to their position in the NCBI 37.3 build map. No additional boundary was added beyond the genes start and stop site. For the autosomal genes the summary statistics from the imputed GWAS were used to derive gene-based statistics using the 1000 genomes (phase 1, release 3) to model linkage disequilibrium. For genes on the X chromosome the genotype data from UK Biobank was used and the gene-based statistic was derived using each participant’s phenotype score. Gene-set pathway analyses were carried out in DEPICT [52] using the genome-wide significant autosomal SNPs as input.

### GWAS of male pattern baldness on a sub-sample of 40,000 and trait prediction in the residual sample of 12,874 participants

For the prediction analysis, the GWAS was re-run on a randomly selected cohort of 40,000 individuals to give regression weights for prediction, leaving an independent cohort of 12,874 in which to test the polygenic predictor. The methods for the GWAS were identical to those reported for the full sample. The regression weights from the GWAS on the 40,000 cohort were used to construct polygenic scores in the target dataset at P value thresholds of <1x10^-20^, <5x10^-8^, <1x10^-5^, <0.01, <0.05, <0.1, <0.2, <0.5, <1 using PRSice software [53]. PRSice creates polygenic scores by calculating the sum of alleles associated with male pattern baldness across many genetic loci, weighted by their effect sizes estimated from the male pattern baldness GWAS. Prior to calculating the scores, SNPs in the prediction dataset were clumped across 250kb sliding windows at an R^2^>0.25. Thereafter, each threshold was used to discriminate between those with no hair loss and those with severe hair loss via logistic regression with results being reported for the optimal predictor only. A predictor for both the autosomes and X chromosome were built and assessed independently and additively. Receiver operator characteristic (ROC) curves were plotted and areas under the curve (AUC) were calculated using the pROC package in R [54, 55].

### Heritability of male pattern baldness

SNP-based heritability of baldness was estimated using GCTA-GREML [56] after applying a relatedness cut-off of >0.025 in the generation of the autosomal (but not X chromosome) genetic relationship matrix.

### Genetic correlations with male pattern baldness

Linkage disequilibrium score (LDS) regression analyses [57] were used to generate genetic correlations between baldness and 24 cognitive, anthropometric, and health outcomes, where phenotypic correlations or evidence of shared genetic architecture have been found (**S7 Table**). Due to the large effects in the *APOE* region for Alzheimer's disease, 500kb was removed from around each side of this region and the analysis was repeated for the Alzheimer's—male pattern baldness analysis. The Alzheimer’s data set without this region is referred to as 'Alzheimer’s 500kb'. In total, we carried out 25 hypothesis tests. Multiple testing was controlled for using a false discovery rate (FDR) correction [58]. An overview of the GWAS summary data for the anthropometric and health outcomes is provided in **S1 Appendix**.

### Partitioned heritability of male pattern baldness

Stratified linkage disequilibrium score (SLDS) regression [59] was used to determine if a specific group of SNPs made a greater contribution to the heritability of male pattern baldness than would be expected by the size of the SNP set. Firstly, a baseline model was derived using 52 overlapping, functional categories. Secondly, a cell-specific model was constructed by adding each of the 10 cell-specific functional groups to the baseline model and the level of enrichment was obtained. Multiple testing was controlled for using FDR correction [58] in both the functional category and cell-specific analysis.

## Supporting information

S1 Table Lookup of GWAS hits from Richards et al. [10], Pickrell et al. [14], Li et al. [15], and Heilmann-Heimbach et al. [16]. (XLSX)

S2 Table Summary information for the 287 independent loci associated with male pattern baldness. (XLSX)

S3 Table Genome-wide significant autosomal gene-based hits (Bonferroni correction of α < 2.769x10^-6^) in the MAGMA gene-based analysis for male pattern baldness. NSNPS is the number of SNPs in the gene.
(XLSX)

S4 Table List of genome-wide significant gene-based hits (Bonferroni correction of α < 8.818x10^-5^) in the MAGMA gene-based analysis for male pattern baldness, performed on the X chromosome. NSNPS is the number of SNPs in the gene.
(XLSX)

S5 Table Results of the Gene-Set Analysis performed in DEPICT. (XLSX)

S6 Table Genetic correlations between baldness and the 24 cognitive, health, psychiatric, and anthropometric variables. The heritability Z-score and the mean χ^2^ indicate the level of power to detect association where a heritability Z-score of >4 and a mean χ^2^ >1.02 being considered well powered [57]. None of the 25 tests performed survived FDR control for multiple comparisons. Nominally significant genetic correlations highlighted in bold. ADHD, attention deficit hyperactivity disorder; MDD, major depressive disorder.
(XLSX)

S7 Table Showing the full output of the partitioned heritability analysis for male pattern baldness. Prop._SNPs refers to the proportion of SNPs from the data set that were a part of the corresponding functional annotation. Statistical significance indicated in bold. Tissue groups are listed in the first ten rows followed by the functional annotation groups.
(XLSX)

S8 Table The 24 health-related phenotypes included in the genetic correlation analysis with male pattern baldness. Verbal-numerical reasoning and childhood intelligence were examined as educational attainment (genetic association with baldness reported by Pickrell et al. 2016 [14]) can be used as a proxy phenotype for general cognitive ability. Metabolic traits were included as metabolic disease has been associated with baldness (references noted in the review paper by Heilmann-Heimbach et al. 2016 [16]). Psychiatric disorders were included due to the association between baldness and neurological conditions such as Parkinson’s disease. Genetic correlations have been observed between baldness and the listed anthropometric and developmental traits [14]. Fertility traits [60] were selected due to the published associations between baldness and the androgen receptor.
(XLSX)

S1 Appendix Sources of GWAS summary results from genome-wide association consortia. (DOCX)

S1 Fig Male pattern baldness QQ Plot for imputed GWAS of autosomal variants (p-values truncated at 1x10^-30^). (PDF)

S2 Fig Enrichment analysis for male pattern baldness using the 52 functional categories in 52,874 individuals. The enrichment statistic is the proportion of heritability found in each functional group divided by the proportion of SNPs in each group (Pr(h^2^)/Pr(SNPs)). Error bars are jackknife standard errors around the estimate of enrichment. The dashed line indicates no enrichment found when Pr(h^2^)/Pr(SNPs) = 1. FDR correction indicated significance at P = 0.011 indicated by asterisk
(TIF)

S3 Fig Enrichment analysis for male pattern baldness using the 10 cell specific functional The enrichment statistic is the proportion of heritability found in 52,874 individuals. In each functional group divided by the proportion of SNPs in each group (Pr(h^2^)/Pr(SNPs). Error bars are jackknife standard errors around the estimate of enrichment. The dashed line indicates no enrichment found when Pr(h^2^)/Pr(SNPs) = 1. FDR correction indicated significance at P = 0.037 indicated by asterisk.
(TIF)
