# Supplementary material for: Genetic prediction of male pattern baldness
Source: PLoS Genet. 2017 Feb 14;13(2):e1006594. doi: 10.1371/journal.pgen.1006594 (PMC5308812; doi:10.1371/journal.pgen.1006594)
Supplement: S1 Appendix — (DOCX) [file pgen.1006594.s009.docx]

**Appendix 1.** Sources of GWAS summary results from genome-wide association consortia.

| **Phenotype** | **URL** | **Reference** | **No. of individuals in GWAS** |
| --- | --- | --- | --- |
| Coronary Artery Disease | http://www.cardiogramplusc4d.org/downloads/ | Schunkert et al., 2011 [[1](#_ENREF_1)] | 22,233 cases  64,762 controls |
|  |  |  |  |
| Type 2 diabetes | http://diagram-consortium.org/downloads.html | Morris et al., 2012 [[2](#_ENREF_2)] | 12,171 cases  56,862 controls |
|  |  |  |  |
| ADHD | https://www.med.unc.edu/pgc/downloads | Cross-Disorder Group of the Psychiatric Genomics Consortium. 2013 [[3](#_ENREF_3)] | 1947 trio cases 1947 trio pseudo controls, 840 cases  688 controls |
|  |  |  |  |
| Alzheimer's disease | http://www.pasteur-lille.fr/en/recherche/u744/igap/igap_download.php | Lambert et al., 2013 [[4](#_ENREF_4)] | 17,008 cases  37,154 controls |
|  |  |  |  |
| Autism | https://www.med.unc.edu/pgc/downloads | Cross-Disorder Group of the Psychiatric Genomics Consortium. 2013 [[3](#_ENREF_3)] | 3,303 cases  3,428 controls |
|  |  |  |  |
| Bipolar disorder | https://www.med.unc.edu/pgc/downloads | Psychiatric GWAS Consortium Bipolar Disorder Working Group. 2011 [[5](#_ENREF_5)] | 7,481 cases  9,250 controls |
|  |  |  |  |
| MDD | https://www.med.unc.edu/pgc/downloads | Major Depressive Disorder Working Group of the Psychiatric GWAS Consortium. 2013 [[6](#_ENREF_6)] | 9,240 cases  9,519 controls |
|  |  |  |  |
| Schizophrenia | https://www.med.unc.edu/pgc/downloads | Schizophrenia Working Group of the Psychiatric Genomics Consortium. 2014 [[7](#_ENREF_7)] | 36,989 cases  113,075 controls |
|  |  |  |  |
| Verbal-numerical reasoning | N/A | Davies et al., 2016 [[8](#_ENREF_8)] | 36,035 |
|  |  |  |  |
| Childhood cognitive ability | http://ssgac.org/Data.php | Benyamin et al., 2014 [[9](#_ENREF_9)] | 17,989 |
|  |  |  |  |
| College degree | http://www.thessgac.org/data | Rietveld et al., 2013 [[10](#_ENREF_10)] | 95,427 |
|  |  |  |  |
| Number of children ever born | http://www.thessgac.org/data | Barban et al., 2016 [[11](#_ENREF_11)] | 343,072 |
|  |  |  |  |
| Age at first birth | http://www.thessgac.org/data | Barban et al., 2016 [[11](#_ENREF_11)] | 251,151 |
|  |  |  |  |
| Obesity | http://www.broadinstitute.org/collaboration/giant/images/9/98/GIANT_EXTREME_BMI_Stage1_Berndt2013_publicrelease_HapMapCeuFreq.txt.gz | Brendt et al., 2013 [[12](#_ENREF_12)] | M = 90,562, SD = 12,476 |
|  |  |  |  |
| HOMA B | <ftp://ftp.sanger.ac.uk/pub/magic/MAGIC_ln_HOMA-B.txt> | Dupuis et al., 2010 [[13](#_ENREF_13)] | 46,186 |
|  |  |  |  |
| HOMA IR | <ftp://ftp.sanger.ac.uk/pub/magic/MAGIC_ln_HOMA-IR.txt> | Dupuis et al., 2010 [[13](#_ENREF_13)] | 46,186 |
|  |  |  |  |
| HbA1c | <ftp://ftp.sanger.ac.uk/pub/magic/MAGIC_HbA1C.txt.gz> | Soranzo et al., 2010 [[14](#_ENREF_14)] | 46,368 |
|  |  |  |  |
| High density lipoprotein cholesterol | [http://www.broadinstitute.org/mpg/pubs/lipids2010/hDL_ONE_Eur.tbl.sorted.gz](http://www.broadinstitute.org/mpg/pubs/lipids2010/hDL_ONE_Eur.tbl.sorted.gz%20) | Teslovich et al., 2010 [[15](#_ENREF_15)] | 99,900 |
|  |  |  |  |
| Low density lipoprotein cholesterol | http://www.broadinstitute.org/mpg/pubs/lipids2010/LDL_ONE_Eur.tbl.sorted.gz | Teslovich et al., 2010 [[15](#_ENREF_15)] | 95,454 |
|  |  |  |  |
| Triglycerides | <http://www.broadinstitute.org/mpg/pubs/lipids2010/TG_ONE_Eur.tbl.sorted.gz> | Teslovich et al., 2010 [[15](#_ENREF_15)] | M = 85,691, SD = 24,764 |
|  |  |  |  |
| Neuroticism | GPC-2.NEUROTICISM.zip | Genetics of personality consortium, 2015 [[16](#_ENREF_16)] | 63,661 |
|  |  |  |  |
| Age at menarche | http://www.reprogen.org/Menarche_Nature2014_GWASMetaResults_17122014.zip | Perry et al., 2014 [[17](#_ENREF_17)] | 132,989 |
|  |  |  |  |
| Waist to hip ratio | http://www.broadinstitute.org/collaboration/giant/images/5/54/GIANT_2015_WHR_COMBINED_EUR.txt.gz | Shungin et al., 2015 [[18](#_ENREF_18)] | 142,475 |

Abbreviations: SSGAC, social science genetic associations consortium; HOMA B, homeostatic model assessment beta-cells; HOMA IR, homeostatic model assessment insulin resistance; HbA1c, glycated haemoglobin; ADHD, attention deficit hyperactivity disorder; MDD, major depressive disorder.

1. Schunkert H, König IR, Kathiresan S, Reilly MP, Assimes TL, Holm H, et al. Large-scale association analysis identifies 13 new susceptibility loci for coronary artery disease. Nature genetics. 2011;43(4):333-8.

2. Morris AP, Voight BF, Teslovich TM, Ferreira T, Segre AV, Steinthorsdottir V, et al. Large-scale association analysis provides insights into the genetic architecture and pathophysiology of type 2 diabetes. Nature genetics. 2012;44(9):981.

3. Cross-Disorder Group of the Psychiatric Genomics Consortium. Identification of risk loci with shared effects on five major psychiatric disorders: a genome-wide analysis. Lancet. 2013;381(9875):1371–9.

4. Lambert J-C, Ibrahim-Verbaas CA, Harold D, Naj AC, Sims R, Bellenguez C, et al. Meta-analysis of 74,046 individuals identifies 11 new susceptibility loci for Alzheimer's disease. Nature genetics. 2013;45(12):1452-8.

5. Sklar P, Ripke S, Scott LJ, Andreassen OA, Cichon S, Craddock N, et al. Large-scale genome-wide association analysis of bipolar disorder identifies a new susceptibility locus near ODZ4. Nature Genetics. 2011;43(10):977-83.

6. Ripke S, Wray NR, Lewis CM, Hamilton SP, Weissman MM, Breen G, et al. A mega-analysis of genome-wide association studies for major depressive disorder. Molecular psychiatry. 2013;18(4):497-511.

7. Schizophrenia Working Group of the Psychiatric Genomics Consortium. Biological insights from 108 schizophrenia-associated genetic loci. Nature. 2014;511(7510):421-7.

8. Davies G, Marioni RE, Liewald DC, Hill WD, Hagenaars SP, Harris SE, et al. Genome-wide association study of cognitive functions and educational attainment in UK Biobank (N=112 151). Mol Psychiatry. 2016. doi: 10.1038/mp.2016.45.

9. Benyamin B, Pourcain B, Davis OS, Davies G, Hansell NK, Brion MJ, et al. Childhood intelligence is heritable, highly polygenic and associated with FNBP1L. Molecular Psychiatry. 2014;19(2):253–8. doi: 10.1038/mp.2012.184. PubMed PMID: 23358156.

10. Rietveld CA, Medland SE, Derringer J, Yang J, Esko T, Martin NW, et al. GWAS of 126,559 individuals identifies genetic variants associated with educational attainment. science. 2013;340(6139):1467-71.

11. Barban N, Jansen R, de Vlaming R, Vaez A, Mandemakers JJ, Tropf FC, et al. Genome-wide analysis identifies 12 loci influencing human reproductive behavior. Nat Genet. 2016;advance online publication. doi: 10.1038/ng.3698.

12. Berndt SI, Gustafsson S, Mägi R, Ganna A, Wheeler E, Feitosa MF, et al. Genome-wide meta-analysis identifies 11 new loci for anthropometric traits and provides insights into genetic architecture. Nature genetics. 2013;45(5):501-12.

13. Dupuis J, Langenberg C, Prokopenko I, Saxena R, Soranzo N, Jackson AU, et al. New genetic loci implicated in fasting glucose homeostasis and their impact on type 2 diabetes risk. Nature genetics. 2010;42(2):105-16.

14. Soranzo N, Sanna S, Wheeler E, Gieger C, Radke D, Dupuis J, et al. Common variants at 10 genomic loci influence hemoglobin A1C levels via glycemic and nonglycemic pathways. Diabetes. 2010;59(12):3229-39.

15. Teslovich TM, Musunuru K, Smith AV, Edmondson AC, Stylianou IM, Koseki M, et al. Biological, clinical and population relevance of 95 loci for blood lipids. Nature. 2010;466(7307):707-13.

16. De Moor MH, Van Den Berg SM, Verweij KJ, Krueger RF, Luciano M, Vasquez AA, et al. Meta-analysis of genome-wide association studies for neuroticism, and the polygenic association with major depressive disorder. JAMA psychiatry. 2015;72(7):642-50.

17. Perry JR, Day F, Elks CE, Sulem P, Thompson DJ, Ferreira T, et al. Parent-of-origin-specific allelic associations among 106 genomic loci for age at menarche. Nature. 2014;514(7520):92-7.

18. Shungin D, Winkler TW, Croteau-Chonka DC, Ferreira T, Locke AE, Magi R, et al. New genetic loci link adipose and insulin biology to body fat distribution. Nature. 2015;518(7538):187-96. doi: 10.1038/nature14132.
