# Supplementary figures and images for: Genetic prediction of male pattern baldness
Source: PLoS Genet. 2017 Feb 14;13(2):e1006594. doi: 10.1371/journal.pgen.1006594 (PMC5308812; doi:10.1371/journal.pgen.1006594)

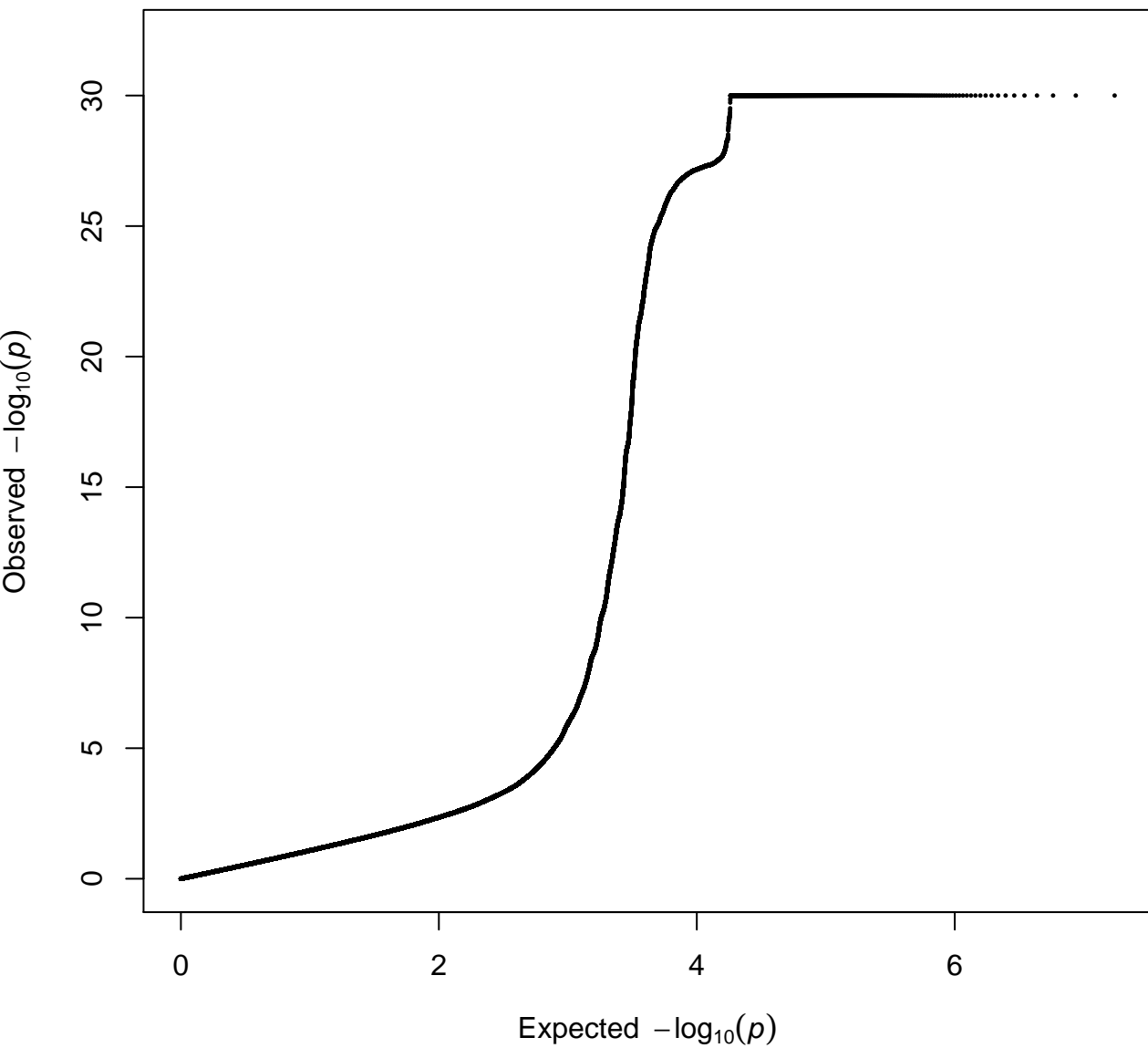

Supplement: S1 Fig — (PDF) [file pgen.1006594.s010.pdf]

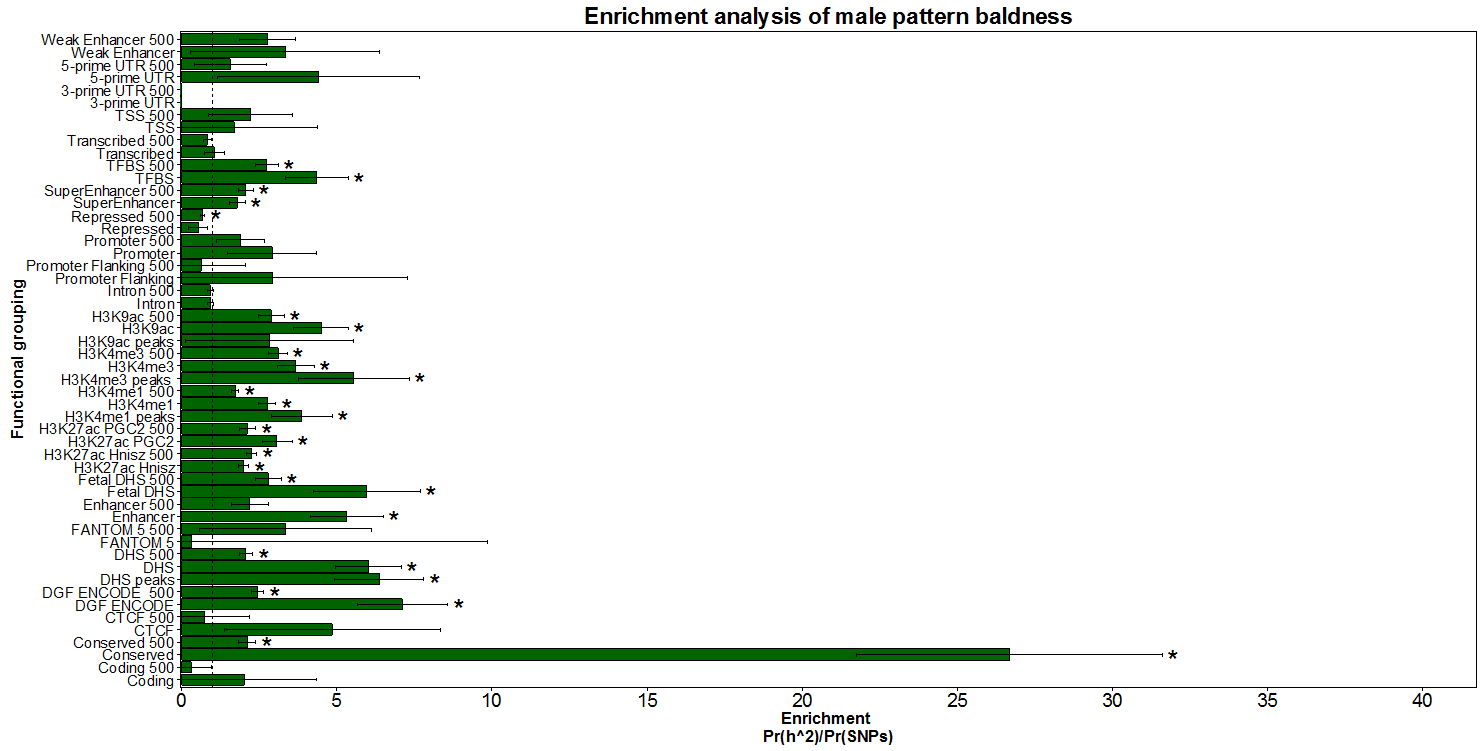

Supplement: S2 Fig — The enrichment statistic is the proportion of heritability found in each functional group divided by the proportion of SNPs in each group (Pr(h2)/Pr(SNPs)). Error bars are jackknife standard errors around the estimate of enrichment. The dashed line indicates no enrichment found when Pr(h2)/Pr(SNPs) = 1. FDR correction indicated significance at P = 0.011 indicated by asterisk (TIF) [file pgen.1006594.s011.tif]

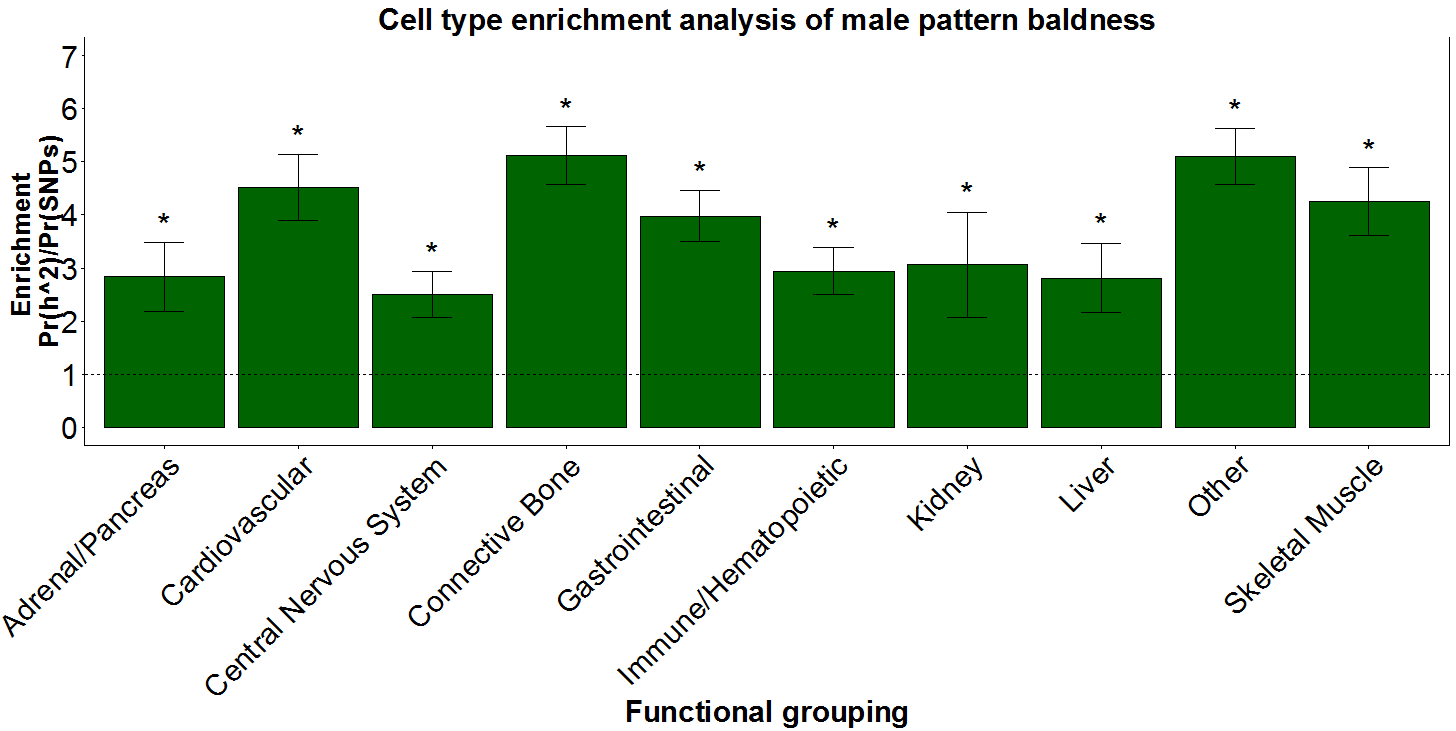

Supplement: S3 Fig — In each functional group divided by the proportion of SNPs in each group (Pr(h2)/Pr(SNPs). Error bars are jackknife standard errors around the estimate of enrichment. The dashed line indicates no enrichment found when Pr(h2)/Pr(SNPs) = 1. FDR correction indicated significance at P = 0.037 indicated by asterisk. (TIF) [file pgen.1006594.s012.tif]
